# Dermoscopy of Facial Angiofibromas in Four Patients of Skin of Color with Tuberous Sclerosis Complex: A Case-Series

**DOI:** 10.5826/dpc.1103a36

**Published:** 2021-07-08

**Authors:** Rashmi Jindal, Sheenam Sethi, Payal Chauhan

**Affiliations:** 1 Department of Dermatology, Himalayan Institute of Medical Sciences, Dehradun; 2 Department of Dermatology, All India Institute of Medical Sciences (AIIMS), Himachal Pradesh

**Keywords:** Facial angiofibroma, angiofibroma, tuberous sclerosis complex, dermoscopy

## Introduction

Facial angiofibromas (FAs) are benign skin tumors seen in 80% of patients with tuberous sclerosis complex (TSC). Full syndrome with skin lesions, mental retardation, and epilepsy with an onset before 5 years of age presents few problems, however there is wide variation in the age of onset and in the severity of skin lesions (angiofibromas, periungual fibroma, shagreen patch, and ash-leaf macules) making the diagnosis difficult. FAs are sometimes overlooked, being confined to the naso-labial folds or chin. Clinical differentiation from trichoepitheliomas, trichofolliculomas, trichilemmomas, dermal melanocytic nevi, acne vulgaris, and syringoma can be challenging, further requiring histopathological examination. Dermoscopy can obviate the need of biospy preventing further disfigurement of the patient’s face. Here we report characteristic dermoscopy findings of FAs in 4 TSC patients of skin of color.

## Case Presentation

**Figure 1:**
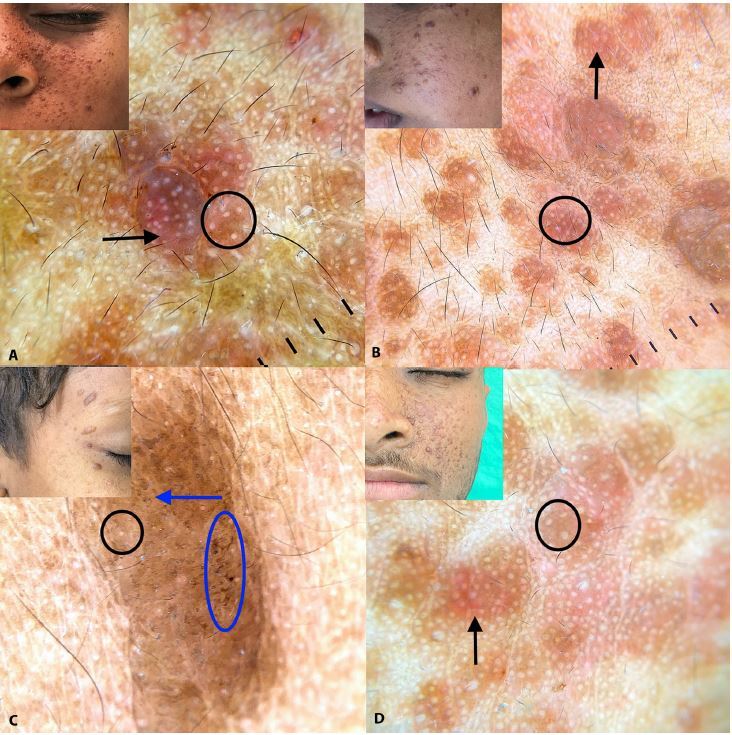
(A–D) Dermoscopy showing yellow-white dots (black circle) over a brown to reddish-brown background (black arrow) with blurred vessels. At places there are unevenly distributed specks/dots of brown pigmentation (blue arrow). Few lesions show surface crypts (blue oval). Inset displays clinical images of the represented dermoscopic findings. (Dermlite©, 3Gen Inc., San Juan Capistrano, CA, U.S.A., DL200 hybrid, Polarized mode, magnification 10x). Images were captured with Dermlite adaptor for Iphone©11.

4 patients reported to skin outpatient department for facial lesions, were diagnosed with TSC, based on the committee of the US National Tuberous sclerosis alliance criteria. All patients had firm, discrete, red-brown papules over the nose, cheeks, and chin. Dermoscopy was performed in all patients, 2 of them, also underwent skin biopsy for histopathological correlation. Dermoscopy findings were similar in all cases and revealed the presence of yellow-white dots over a brown to reddish-brown background with unfocussed vessels. At places there were unevenly distributed specks/dots of brown pigmentation. Few lesions had surface crypts (Figure 1, A–D).

**Figure 2:**
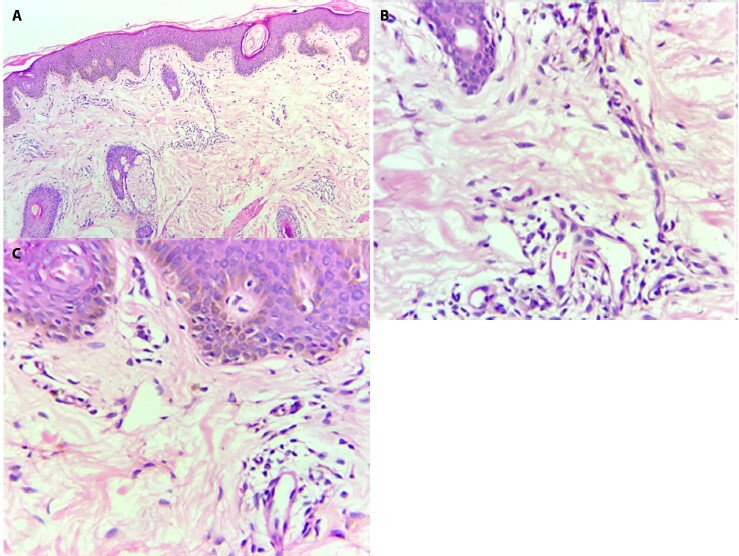
(A) Epidermis showing follicular plugging, patchy to linear melanocytic hyperplasia. Dermis showing increased number of blood vessels surrounded by mild lymphocytic infiltrate. Increased collagen fibers in dermis (H&E, × 10). (B) Increased dermal melanophages (H&E, × 40). (C) Linear melanocytic hyperplasia (H&E, × 40).

Histopathology showed mild hyperkeratosis with follicular plugging, patchy to linear melanocytic hyperplasia, and flattening of rete ridges. Dermis showed an increase in the number of blood vessels with an irregular outline surrounded by mild lymphocytic infiltrate along with pigment incontinence, and prominent dermal melanophages. Increased collagen fibers oriented perpendicular to the surface in sub epidermal zone and around blood vessels and follicles were present (Figure 2, A–C).

## Conclusion

From a dermoscopy-histopathological correlation point of view, yellow-white globules represent follicular hyperkeratosis, diffuse brown pigmentation represents melanocytic hyperplasia, and the background erythema is due to an increased number of dermal blood vessels which also manifest as unfocussed vessels upon dermoscopic examination. Dermal melanophages appear dermoscopically as unevenly distributed dots/specks of brown pigment and surface crypts represent pseudo-follicular openings. Upon dermoscopy, previous investigators have reported findings of facial angiofibromas as yellow-white dots/globules, over a brown-pink, gray background, and red dots in skin of color and appearance of a structureless whitish-pink background reported in phototype I–II [1–3]. We were able to appreciate the uneven brown specks/dots correlating with focally increased dermal melanophages, however red dots were not visualized, instead blurred vessels depicting capillary proliferation were evident.

**Figure 3:**
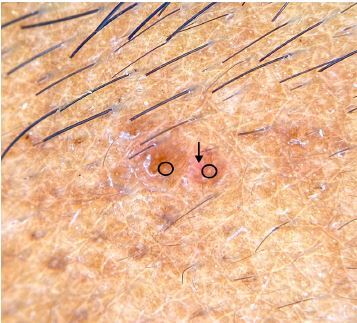
Yellow-white dots (black circle) over a reddish-brown background (black arrow) dermoscopically detected over the pre-auricular area in the undiagnosed father of one of the patients.

Incidentally, 1 of the patients, a 10-year-old boy who did not report a family history, was accompanied by his father, who in turn presented 3–4 brown papules over his right pre-auricular area. The angiofibroma suspect was corroborated by findings upon dermoscopic analysis (Figure 3).

Although FAs seen in TSC are clinically indistinguishable from those present in Birt-Hogg-Dubé syndrome (BHD), facial papules in the latter typically consist of fibrofolliculoma and trichodiscoma as well [4]. Recently, reflectance confocal microscopy on FAs has also revealed regular epidermal honeycombed pattern with nodular structures with no palisading, thick collagen bundles around hair follicles, horizontal linear and vertical round vessels, along with small and angulated bright cells (corresponding to inflammatory cells) [3].

Unilateral facial angiofibromas have been reported and represent a mosaic form of TSC with a tendency of manifestation in the offspring, as occurred in this case. This further strengthens the important role of dermoscopy in the diagnosis of apparently unaffected parents in addition to a full skin examination using Wood’s lamp, ophthalmological examination, and renal ultrasound.
